# Unraveling recombination rate evolution using ancestral recombination maps

**DOI:** 10.1002/bies.201400047

**Published:** 2014-07-14

**Authors:** Kasper Munch, Mikkel H Schierup, Thomas Mailund

**Affiliations:** Bioinformatics Research Centre, Aarhus UniversityAarhus, Denmark

**Keywords:** evolution, genomics, recombination

## Abstract

Recombination maps of ancestral species can be constructed from comparative analyses of genomes from closely related species, exemplified by a recently published map of the human-chimpanzee ancestor. Such maps resolve differences in recombination rate between species into changes along individual branches in the speciation tree, and allow identification of associated changes in the genomic sequences. We describe how coalescent hidden Markov models are able to call individual recombination events in ancestral species through inference of incomplete lineage sorting along a genomic alignment. In the great apes, speciation events are sufficiently close in time that a map can be inferred for the ancestral species at each internal branch - allowing evolution of recombination rate to be tracked over evolutionary time scales from speciation event to speciation event. We see this approach as a way of characterizing the evolution of recombination rate and the genomic properties that influence it.

## Introduction

Recombination is required for proper segregation of homologous chromosomes during the first division of meiosis [1]. Here chromosomes are subjected to programmed double strand breaks [2] and the subsequent 5′ resection creates single strand overhangs that invade the homologous chromosome to form hetero-duplexes [3]. In the majority of cases the following repair occurs by gene conversion but a poorly known proportion results in crossover of chromosomes [4–8]. By means of such crossovers recombination is intimately involved with facilitating adaptive evolution by natural selection, because it shuffles existing variation into new combinations, which allow a species to adapt to environmental challenges. A detailed account of the rate of crossover across the genome is thus of considerable importance for analyses of how evolution has shaped the genomes of current species. In this review we will follow population genetic literature and use recombination rate as a synonym for crossover rate.

The effect of recombination is that the *haploid* genome, which an individual passes on to its offspring, is a combination of the two haploid genomes that the individual received from its parents. Tracing the ancestry of a genome back through time, recombination is seen to split up the genome into small segments that follow distinct lines of ancestry. This change in ancestry along the genome is reflected in the relationship between genomes from individuals of a particular species. In an alignment of such genomes, historical recombination events define segments of the alignment with separate ancestral relationship. This pattern of genetic diversity along the alignment may be summarized by linkage disequilibrium, and used to build recombination maps that describe recombination rate along the genome [9–11]. The recombination rate measured by this approach reflects recombination events accumulating over thousands of generations.

The recombination map over the past few generations can be evaluated from a large number of pedigrees in which individual recombination events are detected by comparison of parent and offspring genomes [12, 13]. An intermediate approach that reveals recombination events within the past 500 years does not require the pedigree to be known, but exploits the observation that African Americans are genetic mixtures of African and European ancestry, and that transitions between African and European ancestry along the genome reflect crossovers [14, 15].

Recombination maps based on these approaches have provided a detailed account of human recombination patterns. Crossovers are not distributed evenly across the genome, and over generations some parts of a genome experience a much higher number of crossovers than others. The majority of recombination is confined to small parts of the genome, in that 60% of crossovers occur in 6% of the genome distributed over roughly 30,000 so-called “hotspots” [11]. These recombination hotspots are less than five thousand bases wide, and exhibit recombination rates that are one to three orders of magnitude larger than that in the surrounding sequence. The location of hotspots is determined by the preference of the PRDM9 protein for certain instances of degenerate DNA motifs [16–19]. This protein mediates recombination events in at least 40% of human hotspots [20] and in practically all hotspots in mouse [21].

The location of hotspots varies among human populations. Many hotspots found in populations from West Africans are absent in European populations because African populations possess variants of the PDRM9 gene not found in Europeans [15]. A fine-scale recombination map based on genetic diversity in western chimpanzees shows that the locations of hotspots in chimpanzees and humans are entirely different [22]. This confirms that hotspots are highly transient [23, 24] with a turnover time of their location shorter than the total divergence time of humans and chimpanzees.

In contrast, the recombination rate at the scale of megabases is remarkably similar among human populations [14, 15] and comparison to chimpanzee has revealed a strong correlation of recombination rate at this scale [22]. The discrepancy between divergence on fine scale and conservation on large scale indicates different forms of control of recombination rate on different scales. The relocation of hotspots to promoter regions in PRDM9 knockout mice [21], and in canines that lack a functional PRDM9 [25, 26], suggests that PRDM9 serves to divert local recombination away from gene promoters where recombination may obstruct transcription.

Apart from the distribution of hotspots the factors producing observed differences in fine-scale patterns of recombination rate between individuals and species are largely unknown. However, differences between species in selective pressures promoting recombination may contribute to such differences, and differences in demography may affect inference of recombination rate. On a megabase scale recombination rate has been correlated with the size of chromosomes [27], chromosomal position [11, 13], strength of crossover-interference [28], sequence GC content [29], transposable elements and repetitive DNA motifs [22], and gene density [30].

So far, detailed recombination maps are only available for yeast [31], fruit fly [32], dog [25, 26], mouse [33, 34], chimpanzee [22], and human [11, 13], but recombination on a chromosomal scale has been compared across mammals and has showed a good correspondence between divergence time and differences in recombination rate [28, 35].

In order to relate evolution of recombination rate to genome evolution we must be able to directly associate genomic change in individual species with the accompanying change in recombination rate. In this review we outline how inference of recombination rate in ancestral species provides such information, and how this may progress our understanding of cause and effect of recombination on the evolution of genomes.

## Tracking evolution of recombination rate

### From divergence to change along individual branches

Differences in recombination rate between two species may result from a change in one of these, or may be the product of change in both. Resolving recombination rate differences into changes that occurred in each species in the course of their divergence requires knowledge of the ancestral recombination rate. However, whereas the ancestral genomic sequence is readily identified by comparison to an out-group using simple models of sequence evolution, the ancestral state of recombination rate cannot be inferred from rates in extant species. To identify change along individual branches of a species tree the recombination rate in the ancestral species must be measured independently.

### Patterns of incomplete lineage sorting reflect ancestral recombination

In the course of time, recombination breaks genomes into small segments with separate ancestry. A consequence of this is that along an alignment of genomes from different species the genealogy will change at each position where one of the genomes has experienced a crossover. This means that ancestral recombination events can be observed as transitions between different genealogical histories along such an alignment. A subset of genealogies has topologies differing from the species tree, and transitions between topologically different genealogies are especially informative about recombination events in ancestral species. Genealogies that differ from the species tree reflect a phenomenon called incomplete lineage sorting (ILS) [36].

**Figure 1:**
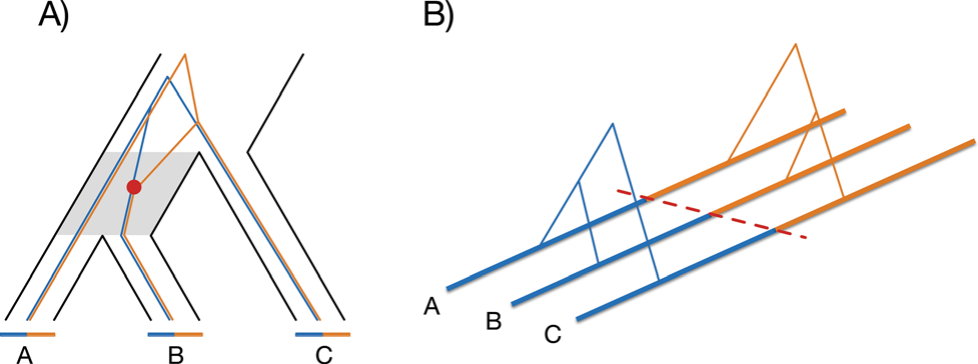
Incomplete lineage sorting reveals ancestral recombination events. **A:** Species tree for three species with lines at the tips representing sequences. A crossover (red dot) in the ancestral species lineage defines two subsequences (orange and blue) with separate genealogies. **B:** Alignment of the three sequences from panel A with underlying genealogies. The red dashed line represents the position of the recombination event.

Tracking one sequence from each of three species backwards in time (Fig. 1A), sequences from the two most closely related species (A and B) may not have found a common ancestor at the time of the speciation event that separates A and B from the third species (C). In this instance the sequence from species C may find a common ancestor with either A or B before these find a common ancestor with each other. If this happens, the tree relating the three sequences will be different from the species tree. In Fig. 1A a recombination event (red dot) in an ancestor to B splits this sequence into two segments (blue and orange) with separate lines of ancestry and a different genealogical relationship to orthologous sequence from the other two species. In the two resulting trees the blue segments of species A and B are most closely related, whereas the orange segments of species B and C are most closely related. The genealogy for the blue segment thus conforms to the species tree, whereas the orange tree presents ILS. Crossovers that mark the transition between such topologies (Fig. 1B) are particularly informative because they primarily occur in the ancestor of the two most closely related species (gray shade in Fig. 1A). ILS occurs when the separate lines of descent for the three species reach back into their common ancestor. This means that ILS is especially common when the time is short between two speciation events and the population size of the intervening ancestral species is large.

### Hidden Markov models can detect ancestral recombination events

**Figure 2:**
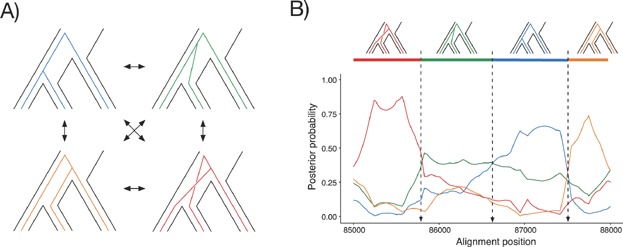
Inference of ancestral recombination events. **A:** The four hidden states of the coalescent HMM which alternate along the alignment. **B:** Posterior probabilities of each hidden state along 3 kb of alignment. The sequence of most likely genealogies is shown at the top.

Coalescent hidden Markov models [37–39] are designed to infer changes in ancestry along an alignment. A hidden Markov model (HMM) is a statistical framework for modeling a Markov chain of observables when the underlying state is unknown (hidden). The columns in the alignment represent the chain of observables, and the hidden states are the underlying genealogies that produce them. The states of the coalescent HMM [37] used by Munch et al. [40], are shown in Fig. 2A. The probability by which each alignment column is produced by the different genealogies in the model is readily computed by posterior decoding of the fitted HMM. Figure 2B shows the probability of each genealogy along 3kb of a genomic alignment of chromosome 2 from human, chimpanzee, and gorilla. Transitions from stronger support of one genealogy to another mark points in the alignment where a crossovers must have occurred in the ancestral species. By recording the position of all such crossovers along a genomic alignment, a recombination map of an ancestral species can be created.

### A map of the human-chimpanzee ancestor

We recently presented a recombination map of the human-chimpanzee ancestor by applying this approach to aligned genomes of human [41], chimpanzee [42] and gorilla [43], and in this way we inferred more than one million ancestral recombination events. As expected, the resulting map corresponds more closely to the human [11] and the chimpanzee maps [22] than these do to each other, even at fine scales (<50 kb). This suggests that the ancestral map possess a resolution comparable to the maps of extant species, despite the different observational base. The recombination events contributing to this map have mainly occurred between the human-chimpanzee and human-gorilla speciation events. The ancestral map therefore describes the average recombination rate on a time scale much longer than recombination maps based on patterns of polymorphism in extant species. Analysis of the ancestral human-chimpanzee map did not identify hotspot motifs, perhaps because their genomic locations have changed several times in the interval of the three million years covered by this map. In addition, whereas polymorphism based maps can measure the effect of more than one crossover between sites, the ancestral map is only able to detect single crossovers that identify the transition between genealogies in the model. Even so, we expect the detected ancestral recombination events to be enriched at hotspots, and this can be used to test whether the hotspot locations have changed between humans, chimpanzees, and their ancestor [40].

### Rate of evolution in recombination rate is not uniform across branches

Comparisons of the recombination maps of human and chimpanzee to that of their ancestor allowed us to evaluate the pace of evolution of recombination in the two species [40]. On both fine and large scales we found that recombination patterns have evolved faster in humans than in chimpanzees. A possible reason is that negative selection conserving recombination patterns is less effective in humans. This would be a consequence of the smaller effective population size in the human lineage [44, 45], where unfavorable mutations would be more likely to replace existing variation and thus contribute to more rapid evolution. It is also possible that the speed at which hotspot locations change is related to population size. Destabilizer-alleles of the hotspot-defining PRDM9 gene influence repeat turnover in the coding sequence of the DNA-contacting zinc-finger array of both alleles in heterozygous males [46]. A higher load of such alleles in a smaller population could potentially contribute to a more rapid turnover of PRDM9 alleles and location of hotspots. The impact of population size on the evolutionary rate of recombination patterns will become clear as more recombination maps of extant species become available for comparison to corresponding ancestral maps.

### Multiple ancestral maps track evolution of recombination rate

**Figure 3:**
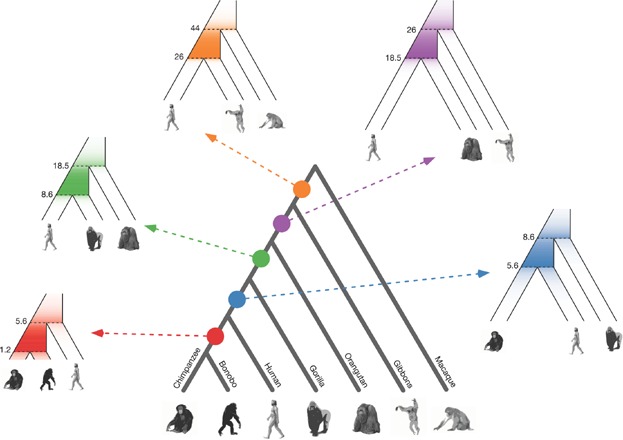
A sequence of hypothetical ancestral recombination maps. Colored dots on the cladogram of old-world monkeys (center) each marks the ancestral species represented by each internal branch. Arrows from each dot point to the species tree of three extant species whose genomes can be used to produce a map of that ancestral species. The color intensity shows the expected distribution of recombination events measured by the constructed recombination map. Numbers represent the speciation times in millions of years used in simulations. Effective population sizes of ancestral species used are: bonobo-chimpanzee: 32,000, human-chimpanzee: 86,000, human-gorilla: 62,000, human-orangutan: 167,000, human-gibbon: 167,000.

Combining multiple ancestral recombination maps allows the evolution of recombination rate to be tracked through a succession of ancestral species. Figure 3 shows the phylogenetic relationship of representatives of old-world monkeys. The speciation events define inner branches that correspond to the ancestral species in the evolution towards chimpanzees (Fig. 1A). Any three extant species that define an inner branch will display ILS, and this allows an ancestral recombination map to be constructed for each of the corresponding ancestral species.

The human-chimpanzee map is the only one completed so far, but we can formulate expectations for the properties of the additional ancestral maps. Following Munch et al. [40] we simulated the coalescent with recombination for one of the species trios that define each inner branch. Speciation times and ancestral population sizes used in our simulations are derived from previous analyses [45, 47, 48], assuming a generation time of 25 years and a constant mutation rate of 6 × 10^−10^ per base pair per year, corresponding to the present day estimate for humans [49–51]. The mutation rate may have changed over the time span covered by ape evolution, and so the choice of this rate most likely overestimates the speciation time and population size of ancestral species [43]. The amount of ILS, however, is not affected by the choice of mutation rate. The effective population sizes of species along external branches are all assumed to be 20,000.

We performed 10,000 simulations for each of the species trios in Fig. 3 and extracted the time of crossovers that contribute to each recombination map. The distribution of crossovers differs between maps as shown by the color intensity along the branches of each species tree. This is a consequence of differences in ancestral population sizes and the time between speciation events. The extent to which contributing crossovers also fall on adjacent branches of the phylogenetic tree is determined by the population size along these branches.

**Figure 4:**
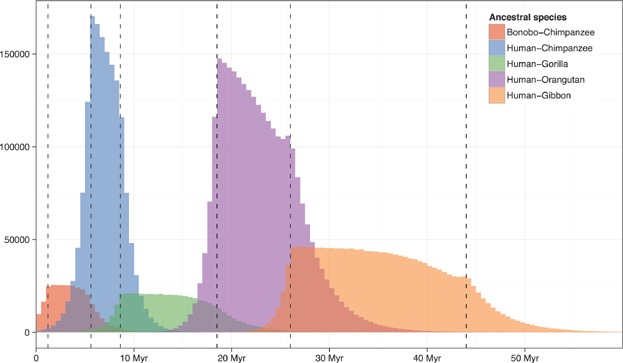
Number of recombination events included in each of the five ancestral recombination maps in Fig. 3 and their distribution in time. Dashed lines represent speciation times.

The power of these maps to measure recombination rate on a fine scale is mainly determined by the amount of ILS between the analyzed extant species, since more ILS will result in more detectable changes to or from a segment with a non-canonical topology. The number and distribution of events contributing to each map is shown in Fig. 4 as overlapping histograms with a bin size of 0.5 Myr. For each map, the distribution of crossovers mirrors those shown in Fig. 3. The total number of events contributing to each map reflects the potential resolution of the recombination map. The human-orangutan map is thus expected to be about as detailed as the published human-chimpanzee map. The human-gibbon map may offer less power, but given the uncertainty of the human-gibbon speciation time, the relative power of the human-orangutan, and the human-gibbon maps may shift to the benefit of the human-gibbon map. The chimpanzee-bonobo and human-gibbon maps may offer less detail, but will still yield estimates of contemporary recombination rate on a broader scale. The simulations demonstrate that our approach is likely to permit the evolution of recombination rates to be tracked through a series of ancestral species. This will allow us to link the rate of change in recombination rate to genomic properties, and to test if recombination rate evolves at a different pace on different branches.

## Genomic determinants of recombination rate

### The hotspot-defining PRDM9 protein evolves rapidly

The architecture of the hotspot-defining PRDM9 protein has been conserved across animal evolution [52]. In contrast, the zinc-finger array responsible for its DNA binding properties evolves very rapidly [52, 53] showing high levels of polymorphism in mice, chimpanzees, and bonobos [54–56]. This rapid evolution may in part result from instability of the zinc-finger tandem repeat structure, driving a high rate of copy number change mutations [46]. However, in both humans, chimpanzees, bonobo, and mice diversity mainly owes to non-synonymous differences encoding the three DNA contacting residues of each zinc-finger [54–56] and positive selection has been found to drive the rapid evolution of these positions in rodents [52].

It has been proposed that positive selection for definition of new hotspots is driven by depletion of current hotspot motifs through biased gene conversion disfavoring DNA motif recognized by PRDM9 [16, 57]. It remains unsettled, however, whether such motif depletion is sufficient to explain the positive selection on PRDM9 [58]. Whatever the mechanism, the rapid evolution of PRDM9 results in a high turnover of alleles and a corresponding short lifetime of individual hotspots.

### Fine scale recombination patterns evolve at an unknown rate

Over the 6–7 million years since the speciation of humans and chimpanzees [43–45] the fine scale recombination patterns have changed so much that recombination hotspots are not shared by the two species, and the recombination map of human-chimpanzee ancestor does not suggest that this ancestor shared hotspots with either humans or chimpanzees [40].

To gauge the rate of turnover of hotspot locations the process must be examined across a much shorter span of time than the divergence of humans and chimpanzees. The newly sequenced genomes of the archaic hominins, the Neanderthal, and the Denisovan [59, 60] offer such an experiment. Humans separated from the ancestor to Neanderthals and Denisovans half a million years ago [59], after which this split into the separate lineages represented by Neanderthals and Denisovans. An alignment of the Neanderthal, Denisovan, and human genomes thus lends itself to construction of an ancestral recombination map that, once compared with current human recombination rate, will reflect changes in the recombination rate on a time scale that is an order of magnitude shorter than that between humans and chimpanzees.

**Figure 5:**
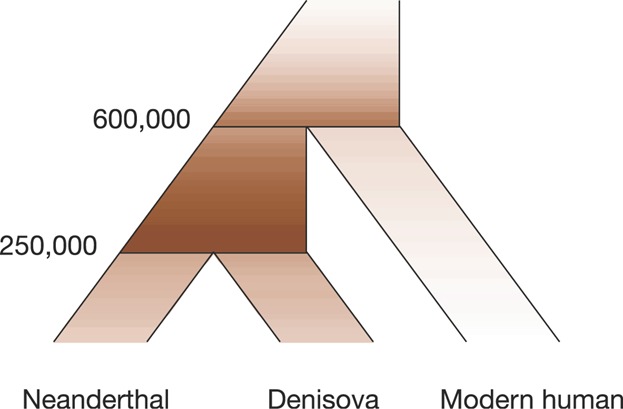
Species tree as in Fig. 3 of Neanderthal, Denisovan, and modern human. Color intensity shows the expected distribution recombination events measured by this hypothetical recombination map. Numbers represent speciation times in years used in simulations. All effective population sizes are set to 10,000.

The details of the demographic processes and speciations of Neanderthals, Denisovans, and humans are not yet known in detail. However, assuming Neanderthal–Denisova speciation 250,000 years ago, Neanderthal-human speciation 600,000 years ago [59], population sizes of 10,000 and a generation time of 25 years, we can simulate the expected distribution of recombination events contributing to a map of the Neanderthal–Denisovan ancestor. The distribution of events shown in Fig. 5 suggests that any differences detected in comparison of this map to the current human map may help elucidate recent evolution of recombination rate.

### Characteristic patterns of recombination in genes

Recombination exhibits characteristic patterns around genes. In humans, promoters show some enrichment of recombination [11], possibly reflecting higher chromatin accessibility of transcriptionally active regions. Exons show substantially lower recombination rates [11], and the recombination rate of introns increases with distance from an exon [13]. Mean recombination rate varies sixfold between ontology-based gene classes [11]. This suggests selection for less recombination in genes with highly conserved function, or selection for higher recombination rate in genes exposed to recurrent adaptive selection for resistance to pathogens. If true, such selective pressures may have changed over evolutionary time, and may be revealed by contrasting recombination rates of different gene ontology classes using the ancestral recombination maps shown in Fig. 3.

### Chromosomal context influences recombination rate

The crossovers that occur in the same meiosis are more evenly distributed over the genome than expected if they were independent [61]. The mechanism by which such crossover-interference is communicated along the chromosome is not known, but one model suggests that mechanical compression stress associated with twisting of the chromatid promotes crossovers and that the release of this stress by a designated crossover reduces the propensity for crossover nearby [61]. In this model crossover-interference also explains crossover homeostasis [62] – the phenomenon that variation in the number of recombination-initiating double strand breaks is not accompanied by a proportionate change in the number of crossovers.

In meiosis each chromosome almost always undergoes at least one crossover [27]. This serves to ensure the physical connection of homologs required for proper segregation and produces a very strong correlation between mean number of crossovers and chromosome length [27, 28]. This “obligatory crossover” of chromosomes may also be a consequence of crossover-interference decreasing variance in number of crossovers per chromosome thus lowering the probability that zero crossovers occur [61].

Distance to chromosome landmarks such as the centromere and telomeres is also correlated with recombination rate. Telomeric regions show higher sex-averaged recombination rates with stronger and more densely spaced hotspots [11] whereas pericentromeric regions show lower recombination rate [13]. The extent of these effects differs among mammalian species and has been associated with strength of crossover interference [28], but it is also hypothesized to result from differences in sequence loop-length in chromatids at the leptotene stage of prophase I [63].

### Rearrangements reveal effect of chromosomal context

Chromosomal rearrangements change the chromosomal context of genomic sequence, and are thus expected to result in changes to local recombination rate.

Nine large inversions have fixed between humans and chimpanzees affecting chromosomes 1, 4, 5, 9, 12, 15, 16, 17, and 18, in addition to the fusion of ancestral chromosomes 2a and 2b into human chromosome 2 [64, 65]. The recombination map of the human-chimpanzee ancestor reveals the consequences of chromosomal rearrangements in the divergence of the two species. In humans a large increase in rate is produced by the inversion spanning the centromere on chromosome 18, and a drastic decrease in rate results from the fusion of chromosome 2. In contrast, the human inversion on chromosome 1 and the eight large inversions in chimpanzee do not have notable effects on the recombination rate. The results of such rearrangements are highly informative of how chromosomal architecture influences large-scale recombination rate because they reveal the effect of different chromosomal contexts on the same genomic sequence.

In contrast to strong karyotype conservation in other apes, gibbons have experienced a very large number of chromosomal rearrangements since their divergence from the great apes [66, 67]. This provides a natural experiment by exposing homologous, and almost identical, genomic sequence to different chromosomal contexts. The four gibbon genera (*Hylobates*, *Hoolock*, *Nomascus*, and *Symphalangus*) show extensive ILS among them [68], and a genome from each genus would thus allow the construction of a recombination map of the common ancestor to gibbons. The corresponding karyotype has been reconstructed [66], and the karyotype of the human-gibbon ancestor can be inferred in the same manner. Comparison of the human map, the ancestral gibbon map and the ancestral human-gibbon map should allow the effect of chromosomal context of the very similar genomic sequence to be quantified in great detail, and this may reveal residual variation controlled by other factors.

### Recombination affects genome evolution

Recombination influences the non-adaptive evolution of genomes by inducing a bias in substitution patterns [69]. In the hetero-duplex regions, produced in both crossover and non-crossover recombination, mismatches are preferentially resolved as GC base pairs in mammals [70, 71]. Such GC-biased gene conversion (gBGC) selects for G or C variants at polymorphic sites in a manner indistinguishable from natural, positive selection. Deleterious GC mutations are thus promoted by gBGC and may fix in the population despite slightly detrimental consequences to organism fitness. Further, aberrant substitution patterns at a specific position in the genome resulting from gBGC may mimic patterns expected from adaptive evolution [72].

As expected from gBGC, the recombination rate is positively correlated with GC-content. However, if recombination rate has changed recently (in evolutionary terms), the GC content will not be at equilibrium, and it will not reflect the potential effect of gBGC. This is described by the equilibrium GC content, GC*, which is easily computed from the substitution rates to and from GC bases [73]. In humans, recombination rate explains 46% of the variance in GC* on a megabase scale [40]. However, recombination maps of living species measure only recent recombination events and are dominated by short-lived hotspots that do not significantly affect substitution rates due to their transient nature [74]. In contrast, evolution of genomic GC-content is a very slow process, and to effectively quantify the effect of gBGC, measures of recombination rate across larger time scales are required.

Ancestral maps allow the effect of recombination rate to be tracked on evolutionary time scales. Recombination rate measured by the human-chimpanzee map represent an average over three million years of recombination (assuming a mutation rate of 6 × 10^−10^) and explains 64% of the variance in GC* on a megabase scale [40]. Considering that the interval where this recombination is measured only accounts for about 25% of the internal genealogical branch where recorded substitutions must have fallen, the true correlation is likely even higher. It is theoretically possible to remove from the recombination map a subset of recombination events that are unlikely to overlap the time interval in which substitutions are counted. This may further increase the correlation with GC* and help quantify the true effect of recombination on genome evolution.

## Conclusions and future directions

The study of recombination and its control has so far focused on individual species to link differences in recombination rate to differences in sequence features in different parts of the genome. This approach has produced much information about how the genome influences recombination. The same approach applied between species does not easily describe how recombination rate evolves and how it interacts with evolution of the genomic sequence. However, ancestral recombination maps produced from comparative analysis of species showing ILS allow differences in recombination rate between species to be resolved into changes along individual branches that can be linked to co-occurring genomic change. Multiple recombination maps also allow evolution of recombination to be tracked through a sequence of ancestors. We believe that inclusion of multiple ancestral maps in comparative analysis of extant species will help advance our understanding of the evolution of recombination rate and its control.

## Abbreviations

gBGC: GC-biased gene conversion
ILS: incomplete lineage sorting
